# Differences in temperament and character inventory (TCI) profile between suicidal and nonsuicidal psychiatric outpatients

**DOI:** 10.1097/MD.0000000000030202

**Published:** 2022-09-02

**Authors:** Sumin Hong, Hong Jun Jeon, Jee Hyun Ha

**Affiliations:** a Department of Psychiatry, Konkuk University Medical Center, Seoul, Korea; b Department of Psychiatry, School of Medicine, Konkuk University, Seoul, Korea

**Keywords:** character, risk assessments, suicide attempt, temperament

## Abstract

The purpose of this study was to identify personality traits associated with suicide attempt in a clinical sample. Temperament and character inventory (TCI) profiles of 759 patients who met the inclusion criteria among 1000 randomly selected hospital records collected. Of these 759 patients, 103 had a history of at least 1 actual suicide attempt (suicidal group) whereas 656 had no such history (nonsuicidal group). The suicidal group showed higher scores of novelty seeking (mean ± SD: 36.1 ± 1.2 vs 33.3 ± 0.5; *P* = .026) and harm avoidance (57.1 ± 1.5 vs 53.0 ± 0.6; *P* = .01) but lower scores of self-directedness (27.5 ± 1.5 vs 34.4 ± 0.6; *P* < .001) than the nonsuicidal group. Higher novelty seeking (OR [95% CI]: 1.031 [1.008–1.054]; *P* = .007) and lower self-directedness: 0.955 [0.927–0.983]; *P* = .002 were also associated with suicide attempts in the analysis of 7 personality scales. These findings suggest that patients who attempt suicide differ from nonattempters in terms of personality traits, especially in novelty seeking (NS), harm avoidance (HA), and self-directedness (SD). It is noteworthy that this study contains data from actual visits to the emergency room to evaluate suicide attempts.

Abbreviations: CO = cooperativeness, Ha = harm avoidance, NS = novelty seeking, PS = persistence, RD = reward dependence, SD = self-directedness, ST = self-transcendence, TCI = temperament and character inventory.

## 1. Introduction

Suicide is an important global public health concern. More than 700,000 people die by suicide every year in the world. Suicide is the fourth leading cause of death among those aged 15–29.^[1]^ The suicide rate in South Korea is the 12th highest in the world and the highest among the Organization for Economic Co-operation and Development (OECD) countries.^[2]^ Although suicide rates have been declining in most OECD countries over the past 20 years, South Korea has consistently maintained a high level of suicide rate (26.9 per 100,000 people in 2019).^[3]^ Therefore, it is imperative to identify the risk factors for suicidal behavior and administer appropriate interventions in clinical practice. A number of bio-psycho-social factors are known to be related to suicidal risk, including psychological factors such as previous suicide attempts, drug and alcohol abuse, depression, anxiety, and other comorbid psychiatric disorders.^[4–6]^ Adverse life events such as a history of abuse, loss of a significant figure,^[7]^ and family conflicts also appear to be social risk factors. In biological aspects, studies have shown that a low function of the serotonergic (5-HT) system might expose individuals to suicidal behavior.^[8–11]^ In clinical practice, suicide attempts are frequently observed after or even without timely problems (depression, loss, substance abuse, etc), suggesting that other significant factors could affect the risk of suicide. It is difficult to identify a single cause of suicidal behavior. Hence, suicide may be due to the interaction between a wide variety of complex causes. In this context, we suspect that personality may be one of underlying risk factors. Even if suicidal patients share similar risk factors, each individual’s behavior is different. Personality traits can influence individual differences. Personality is the product of complex interactions between biological and environmental influences throughout life. It is the basis of immediate or unconscious judgments and actions. Several findings have suggested that personality is formed by both genetic and environment influences.^[12,13]^ In addition, there has been increasing evidence that suicide is associated with underlying specific personality styles.^[14,15]^

Personality is defined as “the dynamic organization within the individual of those psychophysical systems that determine his unique adjustments to his environment”.^[16]^ Although there are various models describing personality, Cloninger model of personality based on a comprehensive psychobiological theory has been widely used for decades.^[13]^ Temperament and Character Inventory (TCI) measures 4 dimensions of temperament [novelty seeking (NS), harm avoidance (HA), reward dependence (RD), and persistence (PS)] and 3 dimensions of personality [self-directedness (SD), cooperativeness (CO), and self-transcendence (ST)], allowing us to understand an individual’s level of maturity, adaptation, and severity of mental illness.^[17]^ Temperaments are defined as automatic associative responses to emotional stimuli that determine habits and moods. They are relatively stable throughout life.^[18]^ Characters defined as “the concept of awareness that affects our voluntary intentions and attitudes” tend to develop constantly as individual’s insights mature into lifelong experiences.^[19]^

Previous studies have shown that suicide attempts are associated with high harm avoidance (HA) and novelty seeking (NS) in mood disorder patients.^[20–22]^ Higher NS and HA scores are also associated with severity of suicidal behavior.^[22]^ An elevated HA score is also positively associated with the number of suicide attempts. It is the strongest and independent risk factor for suicide.^[22]^ A high NS score is related to the lethality of suicide attempts.^[22]^ In the character dimension, low SD is correlated with suicide attempts.^[14,15]^ Other personality traits such as low RD, low CO, and high ST are also associated with suicidal behavior.^[23,24]^ These results suggest that certain personality traits or a combination of traits might have predictive value for an individual’s suicide attempt. As mentioned above, many research studies have already investigated the relationship between personality traits and suicide attempts and some consensus has been reached. However, previous studies had limitations because they were conducted retrospectively based on medical records or personal recalls in a community.^[22,25]^ Recall bias was inevitable in retrospective studies. This could potentially exaggerate the relationship between the risk factors and disease. In addition, some studies were conducted based on local communities,^[26,27]^ which could reduce the homogeneity of the study population. In addition, many studies have been conducted only on subjects with suicide ideation without actual suicide attempts.

This study aimed to compare personality traits between suicidal and nonsuicidal groups in a clinical sample. In this study, about 50% of patients with a history of suicide attempt had a record of visiting the emergency room for suicide attempts. Therefore, suicide attempts were verified. Others were admitted to the Department of Psychiatry within a short period after a suicide attempt. In addition, the study population was a clinical sample that included a control group, ensuring homogeneity of the study subjects.

## 2. Methods

### 2.1. Participants

**Figure 1.**
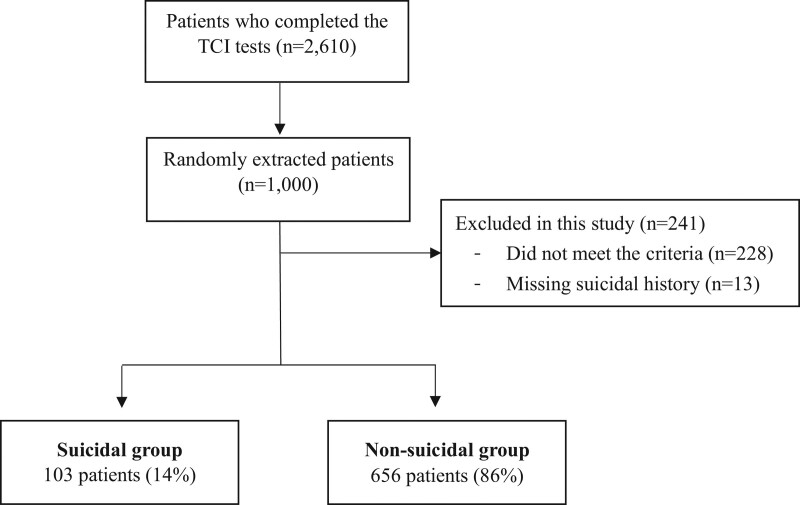
The algorithm used for participants enrollment. Out of the 2610 participants who completed the TCI test, 1000 participants were randomly selected. Based on inclusion criteria, 228 participants were excluded. In total, 759 participants were registered.

Participants who visited the Department of Psychiatry at Konkuk University Hospital between March 2014 and May 2021 were enrolled in this study. The inclusion criteria were: (1) patients who were over 18 years of age; (2) those who had completed the Temperament and Character Inventory (TCI) test; and (3) those who were diagnosed with mood disorders or anxiety disorders. Exclusion criteria were: (1) those who had a history of schizophrenia; (2) those who had a history of manic, hypomanic, or mixed episode; (3) those who currently had psychosis or substance use disorder except alcohol abuse; or (4) those who had other mental disorders with organic causes such as dementia, organic mental disorder, or any other illnesses (medical or neurological). Clinicians used the International Classification of Diseases, 10^th^ revision (ICD-10) to assess and diagnose patients. We randomly extracted 1000 of the 2610 adult patients who completed the TCI test. Of these 1000 individuals, 228 were excluded from the statistical analyses because they did not meet the inclusion criteria. In addition, 13 of them were excluded because suicide attempts were not identified in their medical records. Accordingly, 759 patients were enrolled in the study (Fig. 1). The sample consisted of approximately the same number of male and female participants (male: female = 402: 357), with a mean age of 32.8 years (standard deviation = 12.9). We divided the 759 enrolled patients into 2 groups based on whether they attempted suicide. (number of suicidal group = 103, nonsuicidal group = 656). Patients’ suicide was confirmed by medical record. This study was approved by the Institutional Review Board (IRB) of Konkuk University Hospital (approval number: KUMC 2021-09-017). The requirement for written informed consent was waived by the IRB because of the retrospective nature of the study.

### 2.2. Assessment tools

We used the Temperament and Character Inventory (TCI) to identify temperaments and characters. The TCI included a series of tests developed based on Cloninger psychobiological personality theories.^[19]^ It was designed to measure 4 dimensions of temperament [novelty seeking (NS), harm avoidance (HA), reward dependence (RD), persistence(PS)] and 3 dimensions of character [self-directedness (SD), cooperativeness (C), and self-transcendence (ST)].^[28]^ This study used the Temperament and Character Inventory-Revised Short version (TCI-RS) with verified reliability and validity.^[29]^ The test consists of 140 questionnaires. Individuals were asked to rate each item on a five-point scale. The internal reliability values of the 7 TCI-RS scales ranged from 0.812 to 0.875. In this study, Cronbach α for the TCI subscales was 0.712.

We administered the Beck Depression Inventory-II (BDI-II) and Hamilton Rating Scale for Depression (HAMD) to measure the severity of depressive symptoms. The Korean version of the BDI-II has good internal consistency (Cronbach alpha = 0.89) and test-retest reliability.^[30]^ The HAMD is the most widely used scale by clinicians to assess the severity of depressive symptoms. The Korean version of the HAMD (K-HDRS) showed a high internal consistency (Cronbach alpha = 0.76) and a high inter-rater reliability (*R* = 0.94, *P* < .001).

### 2.3. Statistical analysis

The socio-demographic and clinical characteristics of the patients were analyzed. Summary statistics are presented as means and standard deviations for continuous variables. Numbers and percentages are presented for the discrete variables. The baseline status of education, employment, marriage, clinical diagnosis, family history of suicide attempt, alcohol consumption, and physical disorder were compared between those with a history of suicide attempts and those without a history of suicide attempts using Student t-test. As confounders (age and sex) were excluded by matching, Analysis of Covariance (ANCOVA) was used to explore significant differences in temperament and character dimensions between suicidal and nonsuicidal groups. A logistic regression model was used to identify temperament and personality dimensions that are significant for suicide attempts. All statistical analyses were performed using the Statistical Package for the Social Sciences, version 17 (SPSS Inc., Chicago, IL, USA) with a significance level set at *P* < .05.

## 3. Results

The demographic and clinical characteristics of the 759 patients are summarized in Table 1. There were more female (51.5%) than male (48.5%) participants in the suicidal group, while there were more males (53.7%) than females (46.3%) in the nonsuicidal group. The mean age of participants was 32.8 years. At the education level, most (66.1%) subjects had an under high school education. The suicidal group had a lower proportion of those with more than 12 years of education than the nonsuicidal group (24.3% vs 35.4%, *P* = .022). The suicidal group had a higher proportion of those who were unemployed and unmarried than the nonsuicidal group (unemployed: 68.9% vs 47.7%, *P* < .001; unmarried: 81.5% vs 70.3%, *P* = .015). Depressive disorder NOS (27.7%) was the most common diagnosis, followed by adjustment disorder (22.9%). Of the 759 participants. 9.7% of the suicidal group and 1.5% of the nonsuicidal group had a family history of suicide. Seventy-four (71.8%) participants in the suicidal group had a history of alcohol use, which was higher than that of the nonsuicidal group (50.4%). Of the 759 participants, 10.6% had chronic physical disorder. The suicidal group had more severe depressive symptoms than the nonsuicidal group (BDI-II: mean ± SD = 38.6 ± 12.6 vs 29.1 ± 13.8, *P* < .001; HAM-D: 18.9 ± 6.7 vs 16.5 ± 7.3, *P* = .013). Although not shown in the table, approximately 50% (n = 50) of suicide attempters actually visited the emergency room shortly before the test.

**Table 1:** Socio-demographic and clinical characteristics of participants (n = 759).

	Total sample (n = 759)	Suicidal group (n = 103)	Nonsuicidal group (n = 656)
Sociodemographic characteristics			
Age, mean (SD) years	32.8 (12.9)	29.1 (11.9)	33.4 (13.0)
Gender, N (%) female	357 (47.0)	53 (51.5)	304 (46.3)
Gender, N (%) male	402 (53.0)	50 (48.5)	352 (53.7)
Education status (%)			
<6 years	75 (9.9)	4 (3.9)	71 (10.8)
6–12 years	427 (56.2)	74 (71.8)	353 (53.8)
> 12 years	257 (33.9)	25 (24.3)	232 (35.4)
Employed status, N (%) no	379 (51.0)	71 (68.9)	313 (47.7)
Marital status, N (%) unmarried	545 (71.8)	84 (81.5)	461 (70.3)
BDI-II, mean (SD)	30.4 (14.0)	38.6 (12.6)	29.1 (13.8)
HAM-D, mean (SD)	17.0 (7.3)	18.9 (6.7)	16.5 (7.3)
Clinical characteristics			
Diagnosis, N (%)			
Major depressive disorder	57 (7.5)	25 (24.3)	32 (4.9)
Dysthymia	104 (13.5)	28 (27.2)	76 (11.6)
Depressive disorder NOS	212 (27.7)	21 (20.4)	191 (29.1)
Adjustment disorder	177 (22.9)	19 (18.4)	158 (24.0)
Panic disorder	108 (14.4)	4 (3.9)	104 (15.8)
Others^*^	101 (14.0)	6 (5.8)	95 (14.5)
Family history of suicide, N (%) have	20 (2.8)	10 (9.7)	10 (1.5)
Alcohol consumption, N (%) yes	405 (61.2)	74 (71.8)	331 (50.4)
Physical disorders, N (%) have	80 (10.6)	4 (3.9)	76 (11.6)

* Posttraumatic stress disorder, anxiety disorder nos, insomnia, phobias, somatoform disorder, eating disorder, etc.

**Figure 2.**
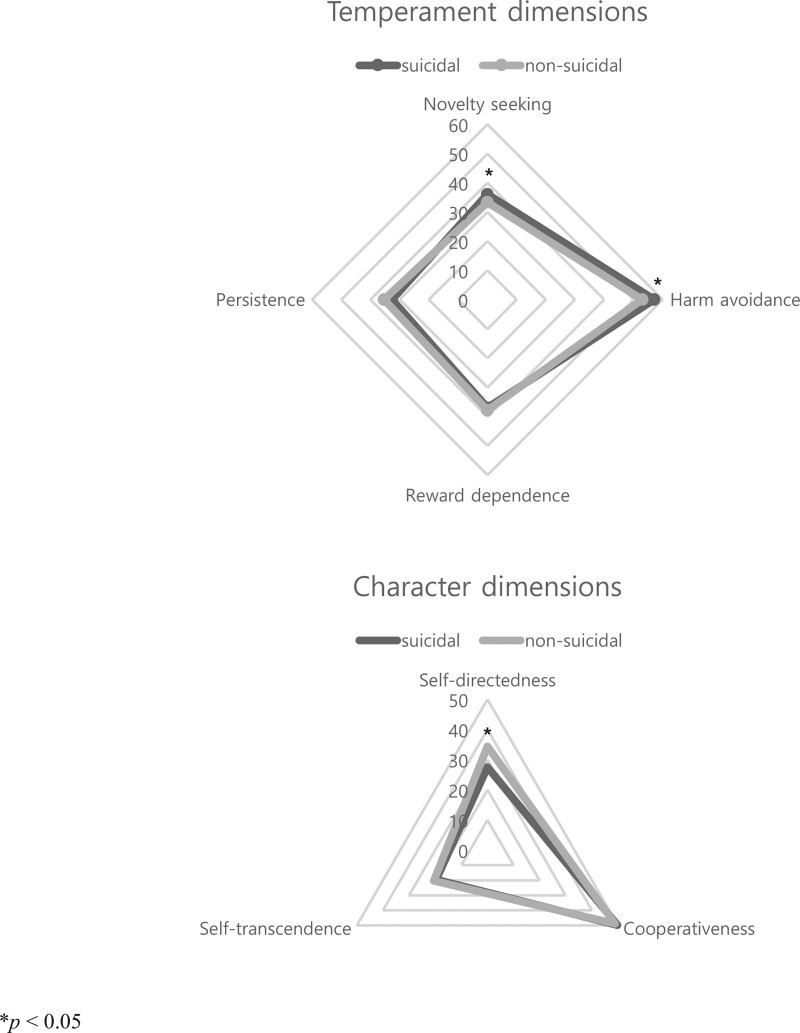
Comparing radar chart of TCI scores between suicidal and nonsuicidal groups. Axes were specified for each temperament and character dimension, and the data were displayed in a polygonal shape over all axes. Suicidal attempters showed higher NS and HA, and lower SD than nonsuicidal attempters.

The results of the comparison of mean personality traits score between the suicidal and nonsuicidal groups are presented in Table 2. Age and sex were adjusted for as covariates. Patients with a history of at least 1 suicide attempt had higher scores of novelty seeking (mean ± SD = 36.1 ± 1.2 vs 33.3 ± 0.5, *P* = .026) and harm avoidance (57.1 ± 1.5 vs 53.0 ± 0.6, *P* = .010) but lower scores of self-directedness (27.5 ± 1.5 vs 34.4 ± 0.6, *P* < .001) than those of nonsuicidal group. A 2-dimensional radar chart (Fig. 2) was created to visualize the differences in the TCI profiles between the suicidal and nonsuicidal groups, as presented in Table 2.

**Table 2:** Comparison of mean scores of 7 personality scales between suicidal and nonsuicidal patients (Analysis of Covariance (ANCOVA) with covariates of age and sex).

	Suicidal group (n = 103)		Nonsuicidal group (n = 656)		
	Mean	Std. error	Mean	Std .error	*P*
Novelty seeking	36.10	1.16	33.31	0.46	**0.026** *
Harm avoidance	57.10	1.48	52.98	0.59	**0.010** *
Reward dependence	36.98	1.27	37.81	0.51	0.542
Persistence	32.62	1.44	35.34	0.57	0.079
Self-directedness	27.51	1.47	34.36	0.58	**< 0.001** *
Cooperativeness	49.80	1.40	48.89	0.56	0.542
Self-transcendence	19.62	1.22	20.52	0.48	0.495

* *P* < .05

A history of suicide attempts was significantly associated with higher NS (OR [95% CI]: 1.031 [1.008–1.054]; *P* = .007) and CO (OR [95% CI]: 1.035 [1.013–1.059]; *P* = .002) but lower scores of SD (OR [95% CI]: 0.955 [0.927–0.983]; *P* = .002) and ST (OR [95% CI]: 0.976 [0.953–0.999]; *P* = .039). (Table 3)

**Table 3:** Coefficients of the model predicting suicide attempt (binary logistic regression model).

Variables	Exp (B)	95% CI	*P*
Novelty seeking	1.031	[1.008, 1.054]	**0.007** *
Harm avoidance	0.994	[0.968, 1.022]	0.684
Reward dependence	0.985	[0.963, 1.007]	0.177
Persistence	0.998	[0.976, 1.021]	0.861
Self-directedness	0.955	[0.927, 0.983]	**0.002** *
Cooperativeness	1.035	[1.013, 1.059]	**0.002** *
Self-transcendence	0.976	[0.953, 0.999]	**0.039** *
(Constant)	0.172		0.212

CI = confidence interval for Exp (B).

* *P* < .05.

## 4. Discussion

In this study, higher NS and HA but lower SD traits were associated with suicide attempts. There were no significant group differences in RD, P, CO, or ST scores between the 2 groups. These results were similar to those of previous studies conducted on community samples or nonclinical subjects.^[15]^ Of 759 participants, 13.6% had a lifetime history of at least 1 suicide attempt. Female sex, lower education level, and unmarried status were associated with attempted suicide. In terms of diagnosis, there were more major depressive disorder and dysthymia but fewer depressive disorder NOS, adjustment disorder, panic disorder, and others in the suicidal group than in the nonsuicidal group. In the case of depressive symptoms assessed by BDI-II and HAM-D, more severe depressive symptoms were associated with suicidal behaviors, regardless of the diagnosis.

Several previous studies have found that depressive symptoms are correlated with suicidal ideation and that such symptoms could accurately predict suicidal ideations.^[31,32]^ However, due to the social and biological complexity of suicide, the association between depressive symptoms and suicidal behavior remains unclear. In actual clinical trials, we observed that suicide attempts occurred frequently even after depressive symptoms had almost disappeared, requiring a supplementary explanation. On the contrary, some people did not attempt suicide despite a severe degree of depressive symptoms. Other background factors may also affect the risk of suicide attempts. Temperament and personality aspects could be major factors explaining this unclear phenomenon.

In the current study, higher NS and HA and lower SD were associated with suicide attempts. NS is defined as a tendency toward repetitive search activities in response to unfamiliar stimuli, impulsive decision-making, rapid loss of temper, and active avoidance of frustration.^[18]^ Those with high NS scores are described as impulsive, exploratory, wasteful, and somewhat irritable. Those with high HA scores are shy, pessimistic, fearful, and easily exhausted.^[33]^ Many studies have shown that high HA scores are associated with suicidal risk,^[15,23,26]^ as well as the severity and frequency of suicide attempts.^[22]^ In our study, the correlation between HA and suicide attempts was significant. However, the severity of the suicide method and frequency were not significantly related to suicide attempts. SD is a personality characteristic of self-determination which is the ability to adjust and adapt behavior according to the demands of a situation to achieve personal goals and values.^[19]^ A low SD is related to the tendency to take a responsibility for actions or decisions and to blame others. Those with low SD can drift for no purpose in life, have significantly poor problem-solving skills, and lack confidence in their self-efficiency. Therefore, low SD is associated with many problems in life. It is highly correlated with many personality disorders.^[34]^ Our suicide attempters showed significantly lower SD scores, consistent with previous findings.^[19,34]^

From a personality perspective, HA is regarded as a common biogenetic background of intrinsic mental illnesses such as depression and anxiety disorders. We assume that high HA alone does not increase the risk of suicide. The risk increased only when high HA and NS levels were existed together. This is because NS is associated with impulsivity and extreme decision.^[18]^ In the character dimension, a lower SD was associated with introversion and inhibition of emotional expression. Subjects with lower SD might have been experiencing depressive mood for a long time without appropriate expression of their discomfort. Eventually, at some point, an individual can decide to attempt suicide as an extreme and impulsive act. In summary, we cautiously propose that the interactions of these 3 personality factors (HA, NS, and SD) might play a major role in an individual’s actual suicide attempt.

This study had several limitations. First, because it was a self-report study in which the analysis relied only on answers given by patients, this study may have some validity issues. Second, the methodology of the TCI research had several limitations. For example, the reliability of harm avoidance has been questioned as it may depend on psychopathology or diagnostic status. High scores of harm avoidance are associated with various mental disorders such as depression, bipolar disorder, eating disorders, OCD, panic disorders, and so on.^[33,35]^ Third, the results of our study cannot be directly applied to the general population since all subjects were clinical samples. In addition, this study did not use structured diagnostic tools at the time of patient registration. Although the use of an appropriate diagnostic tool in research design is important, we did not focus on the accuracy of the diagnostic name. This is because the main purpose of this study was to determine the personality traits in the patients with suicide attempts, especially within the clinical sample. Therefore, we considered that the ICD-10^th^ was sufficient for diagnosis in this study. We also tried to find the difference in TCI according to the number and severity of suicide between the suicide attempters and the nonsuicide attempters. However, no significant differences were observed.

The advantage of this study was that we included many suicide attempters identified through emergency room visits. Most retrospective studies have confirmed suicide attempts through questionnaires or interviews. However, in our study, more than 50% of the participants had visited the emergency room of this institution with suicide attempts as the chief complaint. Therefore, our subjects can be viewed as having more accurate and lively data than those in other retrospective studies. In addition, since our study was a clinical sample mainly focusing on depression, it targeted a more homogeneous group compared to studies analyzing community samples. In addition, considering the genetic and neurobiological basis, TCI has a great advantage in that it can be used to understand patients not only in diagnostic conditions such as depression, but also in biogenetic aspects. This has great implications in that regardless of diagnosis, the actual risk of suicide can be evaluated by evaluating temperaments and characteristics in a clinical setting.

In conclusion, patients with a history of suicide attempts showed different personality and temperament patterns than nonsuicidal participants. Our results emphasize the intrinsic contribution of high novelty-seeking (NS), harm-avoidance (HA), and low self-directedness (SD) to suicidal behavior. Therefore, the early detection and intervention of clinical patients with a TCI profile characterized by high NS, HA, and low SD can reduce the prevalence of suicidal behavior.

## Author contributions

Sumin Hong: Data curation, Formal analysis, Writing – original draft, visualization

Hong Jun Jeon: Formal analysis, Methodology, Writing - original draft

Jee Hyun Ha: Conceptualization, Supervision, Writing – review & editing
